# Enhancing Human Cognition with Cocoa Flavonoids

**DOI:** 10.3389/fnut.2017.00019

**Published:** 2017-05-16

**Authors:** Valentina Socci, Daniela Tempesta, Giovambattista Desideri, Luigi De Gennaro, Michele Ferrara

**Affiliations:** ^1^Department of Biotechnological and Applied Clinical Sciences, University of L’Aquila, L’Aquila, Italy; ^2^Department of Life, Health and Environmental Sciences, University of L’Aquila, L’Aquila, Italy; ^3^Department of Psychology, “Sapienza” University of Rome, Rome, Italy

**Keywords:** chocolate, flavanols, neuroprotection, cognitive function, cardiovascular function

## Abstract

Enhancing cognitive abilities has become a fascinating scientific challenge, recently driven by the interest in preventing age-related cognitive decline and sustaining normal cognitive performance in response to cognitively demanding environments. In recent years, cocoa and cocoa-derived products, as a rich source of flavonoids, mainly the flavanols sub-class, have been clearly shown to exert cardiovascular benefits. More recently, neuromodulation and neuroprotective actions have been also suggested. Here, we discuss human studies specifically aimed at investigating the effects of acute and chronic administration of cocoa flavanols on different cognitive domains, such as executive functions, attention and memory. Through a variety of direct and indirect biological actions, in part still speculative, cocoa and cocoa-derived food have been suggested to possess the potential to counteract cognitive decline and sustain cognitive abilities, particularly among patients at risk. Although still at a preliminary stage, research investigating the relations between cocoa and cognition shows dose-dependent improvements in general cognition, attention, processing speed, and working memory. Moreover, cocoa flavanols administration could also enhance normal cognitive functioning and exert a protective role on cognitive performance and cardiovascular function specifically impaired by sleep loss, in healthy subjects. Together, these findings converge at pointing to cocoa as a new interesting nutraceutical tool to protect human cognition and counteract different types of cognitive decline, thus encouraging further investigations. Future research should include complex experimental designs combining neuroimaging techniques with physiological and behavioral measures to better elucidate cocoa neuromodulatory properties and directly compare immediate versus long-lasting cognitive effects.

## Introduction

With the increase in general population aging, age-related cognitive decline has progressively become a major public health issue. Consequently, enhancing cognitive resources came to be a priority scientific challenge, mainly driven by the need to detect preventive agents for cognitive decline and dementia. At the same time, immediate cognitive enhancement is often desirable in cognitively demanding environments. Stimulants such as caffeine are indeed commonly used to sustain cognitive performance during periods of fatigue and prolonged wakefulness (1).

In the last years, the field of nutraceuticals and functional food has developed along with the interest in the potential modulatory effects of food constituents on human health. In this context, there has been increasing interest in the biological effects of flavonoids, a class of polyphenolic compounds, as potential nutraceuticals with several beneficial biological actions, including cardioprotection, neuroprotection, and neuromodulation. Particularly, cocoa bean has been recognized as a rich source of flavonoids, mainly the flavanols subclass in the form of epicatechin and catechin (2).

Chocolate also contains other functional ingredients, such as the methylxanthine caffeine and theobromine, with the potential to influence neurocognitive function. However, relative to total flavanols content, caffeine and theobromine concentrations in cocoa and chocolate have been considered lower than those required to exert significant pharmacological actions (3).

Epidemiologic studies suggest that a regular intake of flavonoids could be associated with better cognitive function (4), decreased risk of dementia (5) and cognitive decline (6), lower prevalence of cognitive impairment (7), better cognitive evolution over a 10-year period (8), and better dose-dependent cognitive performance in normal aging (9).

The specific mechanisms of action of flavonoids responsible for cognitive protection and modulation are not entirely elucidated. Nevertheless, increasing evidence supports the notion that cocoa and chocolate consumption provides several health benefits, including neurocognitive enhancement and neuroprotective effects. In this brief review, we discuss human studies specifically aimed at investigating the effects of acute and chronic administration of cocoa flavanols on cognition.

## Enhancing Cognition with Cocoa Flavonoids: Direct Versus Indirect Mechanisms of Action

The beneficial effects of polyphenolic compounds have been first attributed to their ability to exert antioxidant actions (10). However, due to the very low concentration of flavonoids detectable in the brain, it is unlikely that direct antioxidant action can entirely account for cognitive effects *in vivo*. Instead, flavonoids’ neurobiological effects are now believed to be mediated by a range of actions involving the ability to protect vulnerable neurons, enhance neuronal function, and stimulate regeneration *via* interaction with neuronal intracellular signaling pathways involved in neuronal survival and differentiation, long-term potentiation (LTP), and memory (11).

Flavonoids can counteract neuronal injury underlying neurodegenerative diseases such as Parkinson and Alzheimer diseases through their interaction with signaling proteins important in the pro-survival pathways (12). Interestingly, flavonoids and their metabolites cross the blood–brain barrier and have been localized in the brain, particularly in areas crucial for learning and memory such as hippocampus, cerebral cortex, cerebellum, and striatum (13–15). These structures are particularly vulnerable to the effects of aging and neurodegeneration, suggesting that flavonoids could exert direct neuroprotective effects (16). Direct interactions within several cellular signaling pathways have been described, such as mitogen-activated protein kinase, extracellular signal-regulated kinase, and phosphoinositide 3-kinase (PI3-kinase/Akt) signaling cascades (17), that are crucially involved in triggering gene expression and protein synthesis for LTP (18). In memory-related areas such as the hippocampus, flavonoids promote the expression of brain-derived neurotrophic factor (BDNF), that is crucial to adult neurogenesis, synaptic growth, and neuronal survival (19). Increasing evidence from animal models indicates that flavonoids can promote cognitive benefits through their ability to directly interact with the cellular and molecular architecture involved in memory function (2, 11, 12). To date, however, it is still not entirely clear whether and to what extent these direct biological actions also extend to the human brain.

Flavonoids could also display their neurocognitive effects indirectly, through their capability of inducing cardiovascular actions. In this regard, cardiovascular benefits of cocoa and chocolate consumption are now well established and include endothelium-dependent vasodilation, which contributes to the maintenance of normal blood flow, reduced platelet aggregation, and blood pressure improvement (20–26).

Importantly, flavanols such as epicatechin can improve endothelial function by increasing the nitric oxide (NO) bioavailability, a key regulator of vascular function, leading to improvements in vascular tone and blood pressure regulation (27, 28). These peripheral vascular changes may also extend to brain perfusion, leading to a more efficient cerebrovascular coupling during neuronal activation, which is considered crucial for the functional and structural integrity of the brain. Moreover, as increased cerebrovascular function promotes adult neurogenesis in the hippocampus (29), flavonoid-mediated vascular changes could be additionally relevant for memory function.

Coherently, increasing evidence highlights the potential of cocoa flavanols to influence cognitive abilities interacting with the cerebrovascular system. Flavanol-rich cocoa has been shown to significantly increase cerebral blood flow (CBF) 1–2 h post-intervention in humans (30–32), suggesting that flavanols’ indirect, vasodilatatory actions could account for acute cognitive enhancement following a single administration (16). Conversely, cognitive benefits associated with long-term flavonoids intake are more likely to involve morphological changes induced by direct actions on neuronal signaling. However, indirect mechanisms of action can also contribute to long-term neuroprotective effects, as increases in NO levels can further impact on brain vascularization over time by promoting angiogenesis, thus resulting in better cerebrovascular communication.

## Effects of Cocoa Flavanols Daily Intake on Cognition: Evidence from Chronic Investigations

In line with the evidence suggesting a favorable association between flavonoids’ regular consumption and cognitive performance in elderly subjects (33), chronic and sub-chronic cognitive effects of cocoa flavanols administration have been addressed in normal aging and clinical populations (see Table 1).

**Table 1:** **Summary of the studies examining the effects of daily administration of cocoa flavanols on cognitive performance**.

Reference	Participants	Flavanols amount	Cognitive measures	Principal findings
Francis et al. (31)	16 subjects (all females; 18–30 years)	Cocoa drinks containing 172 and 13 mg flavanols daily over a 5-day period	Task switching paradigm	Increased BOLD signal in response to the task switching paradigm after the higher flavanol drink, with no significant behavioral effect
Crews et al. (34)	101 subjects (41 males, 60 females; mean age: 69 years)	Chocolate bar containing 397 mg flavanols, chocolate drink containing 357 mg flavanols, or similar placebo	Selective Reminding Test, Stroop Test, Trail Making Test, Wechsler Adult Intelligence Scale-III (Digit Symbol-Coding Subtest), Wechsler Memory Scale-III (Faces I and Faces II Subtests)	No differences in cognitive performance between placebo and polyphenol groups
Camfield et al. (35)	63 subjects (40–65 years)	Cocoa drinks containing 500, 250, and 0 mg flavanols (placebo) over a 30-day period	Spatial Working Memory Test	SSVEP changes indicating improved working memory function after flavanol treatment, with no significant behavioral effect
Desideri et al. (36)	90 subjects with Mild Cognitive Impairment (43 males, 47 females; 64–82 years)	Cocoa drinks containing 993, 520, or 48 mg (low-flavanol) flavanols daily over an 8-week period	Mini-Mental State Examination, Trail Making Test (A–B), Verbal Fluency Test	Improvements in blood pressure, insulin resistance and Tail Making Test (A–B) performance for high and intermediate flavanol groups compared to the low-flavanol group. Improved verbal fluency performance in the high-flavanol group
Sorond et al. (37)	60 subjects with vascular risk factors (29 males, 31 females; mean age: 72.9 years)	Cocoa drinks containing 609 or 13 mg (flavanol-poor) flavanols daily over a 30-day period	Mini-Mental State Examination, Trail Making Test (A–B)	Improvements in neurovascular coupling and Trial Making Test B performance for the flavanols group, only in those with impaired neurovascular coupling at baseline
Brickman et al. (38)	37 subjects (13 males, 24 females; 50–69 years)	Cocoa supplement containing 900 or 10 mg (low-flavanol) flavanols daily over a 3-month period	Modified Benton Task (dentate gyrus-dependent memory task)	Correlation between increased cerebral blood volume in the dentate gyrus and improvements in the Modified Benton Task performance in the high-flavanol group
Mastroiacovo et al. (39)	90 subjects (37 males, 53 females; 65–85 years)	Cocoa drinks containing 993, 520, or 48 mg (low-flavanol) flavanols daily over an 8-week period	Mini-Mental State Examination, Trail Making Test (A–B), Verbal Fluency Test	Improvements in blood pressure, insulin resistance and Tail Making Test (A–B) performance for high and intermediate flavanol groups in comparison to low-flavanol group. Improved verbal fluency performance among all treatment groups
Neshatdoust et al. (40)	40 subjects (22 males, 18 females; 62–75 years, mean age: 68.3 years)	Cocoa drinks containing 494 and 23 mg (low-flavanol) flavanols daily over a 28-day period	Several cognitive tasks measuring executive functions, episodic memory, working memory, spatial memory, implicit memory, attention and processing speed	Higher BDNF serum levels and improvements in global cognition scores following high-flavanol treatment

*BDNF, brain-derived neurotrophic factor; BOLD, blood oxygenation level-dependent; SSVEP, steady-state visually evoked potentials*.

In this respect, the daily consumption of flavanol-rich cocoa drink has been showed to positively affect cognition, leading to improvements in cognitive performance both in older adults with early memory decline (36) and in cognitively intact elderly subjects (39). Specifically, compared to the low-flavanol condition (48 mg), the chronic administration of intermediate (520 mg) and high (993 mg) cocoa flavanols content over an 8-week period was associated to improvements in processing speed, executive function, and working memory in subjects with mild cognitive impairment. At higher cocoa flavanols concentrations, significant improvements were also evident in a verbal fluency task. Interestingly, such cognitive beneficial effects were paralleled by improvements in blood pressure and insulin resistance, suggesting a role of endothelial function and glucose sensitivity in modulating cognitive function in these patients (36). More recently, similar findings were replicated in healthy aged participants. Subjects in the intermediate and high cocoa flavanols groups, after a daily consumption over an 8-week period, showed better performance in several cognitive domains compared to those in the low-flavanol group (39).

By contrast, in healthy older adults a 6-week cocoa flavanols intervention showed no significant effects on cognitive and cardiovascular outcomes (34). In this study, however, participants were administered chocolate bar or beverage containing 397 or 357 mg flavanols, respectively; insufficient flavanols content may therefore account for the negative finding.

In middle-aged (40–65) subjects, Camfield et al. (35) investigated the chronic (30-day intake) neurocognitive effects of 250 and 500 mg cocoa flavanols administration using a spatial working memory task and steady-state visually evoked potentials (SSVEP). Compared to placebo, participants receiving cocoa flavanols treatment showed changes in SSVEP average amplitude and phase across several posterior parietal and centro-frontal sites that indicated an increased neural efficiency in response to the working memory task. Hence, cocoa flavanols long-term intake could effectively improve cognition trough increased neural efficiency, although, in this healthy population, the observed changes in brain activation were not paralleled by concomitant improvements in spatial working memory accuracy. Similarly, a sub-chronic (5 days) daily intake of 172 mg cocoa flavanols was associated, in healthy young subjects, to increased blood oxygenation level-dependent signal in various brain regions in response to an attention switching task, without any behavioral effect (31).

It has been proposed that cocoa long-term cognitive protection could particularly affect populations at risk. In this regard, Sorond et al. (37) evaluated the effect of flavanol-rich cocoa on neurovascular coupling in older subjects with vascular risk factors. In patients with impaired cerebrovascular coupling at baseline, cocoa administration was associated with improvements in cerebrovascular function both acutely and after a 30-day period. Moreover, chronic flavanols intake also resulted in improvements in cognitive flexibility. Conversely, no significant effects of cocoa administration on neurovascular coupling and cognitive performance were observed in those with intact cerebrovascular functions (37).

Besides improving CBF and CBF velocity after daily intake (41, 42), cocoa flavanols could also specifically affect cerebral blood volume in memory-related brain areas (38). Using high-resolution functional magnetic resonance imaging, a 3-month intervention with 900-mg cocoa flavanols resulted in increased cerebral blood volume in the dentate gyrus of the hippocampus, a structure particularly affected by aging and potentially implicated in memory decline. Moreover, the increase in cerebral blood volume was highly correlated with improvements in performance in a dentate gyrus-dependent memory task. Cocoa flavanols regular intake could therefore improve human dentate gyrus function through global increase in blood flow or a more selective increase in capillary density.

More recently, Neshatdoust et al. (40) investigated the link between cognitive performance and BDNF serum levels in older participants following cocoa flavanols chronic administration over 12 weeks. Results showed that, in comparison to a low-flavanol control (23 mg total flavanols), the daily intake of a high-flavanol cocoa drink (494 mg total flavanols) was associated with higher BDNF serum levels and improvements in global cognition scores. Therefore, changes in serum BDNF levels induced by cocoa flavanols could additionally underpin the beneficial effect of chronic cocoa flavanols intake on cognitive functions.

Collectively, these findings seem to support quite consolidated epidemiological evidence indicating that regular cocoa flavanols intake possesses the potential to protect human cognition, particularly in aged populations (33). As suggested (43), cocoa flavanols long-term beneficial effects on cognition could also possibly explain the correlation between country levels of chocolate consumption and Nobel Prizes *per capita*.

## Acute Effects of Cocoa Flavanols Intake on Cognition

Only few randomized controlled trials have investigated the possibility that human cognitive function may be improved following acute cocoa consumption, within 6 h post-ingestion (see Table 2).

**Table 2:** **Summary of the studies examining the effects of acute administration of cocoa flavanols on cognitive performance**.

Reference	Participants	Flavanols amount	Cognitive measures	Principal findings
Scholey et al. (44)	30 subjects (13 males, 17 females; 18–35 years)	Dairy based cocoa drinks containing 994, 520, and 46 mg flavanols (control)	Rapid Visual Information Processing Task, Serial Threes Subtraction, Serial Sevens Subtraction	Improvements in Serial Threes performance following 994 and 520 mg flavanols compared to 46 mg. Improved visual information processing performance after 994 mg flavanols
Field et al. (45)	30 subjects (8 males, 22 females; 18–25 years)	Dark chocolate containing 773 mg flavanols and white chocolate (control)	Choice Reaction Time, Visual Spatial Working Memory Test	Improvements in spatial working memory performance and choice reaction time in the flavanol condition
Pase et al. (46)	71 subjects (40–65 years; 34% males)	Chocolate drinks containing 500, 250, or 0 mg flavonoids (placebo)	Mood assessment, Cognitive Drug Research Battery measuring working memory, episodic memory, speed of memory and attention	No differences in cognitive performance between placebo and treatment groups
Massee et al. (47)	40 subjects (18–40 years; 16% males)	Cocoa bars containing 250 or 0 mg flavanols (placebo)	Mental fatigue assessment, Serial Threes and Serial Sevens, Rapid Visual Information Processing Task	Improvements in serial sevens subtraction performance and self-reported mental fatigue in the flavanol group. No significant effect for cardiovascular measures
Decroix et al. (48)	12 subjects (all males; mean age: 30 years)	Cocoa drinks containing 903 and 15 mg flavanols (placebo)	Stroop task	Increased cerebral blood oxygenation (NIRS) during the task with no significant behavioral effect
Grassi et al. (27, 28)	32 subjects(16 males, 16 females; mean age: 25.3 years)	Chocolate bars containing 520 mg (flavanol-rich) and 88.5 mg flavanols (flavanol-poor)	Psychomotor Vigilance Task, 2-back task	Improvements in 2-back task accuracy after flavanol-rich treatment following a night of total sleep deprivation, in females. In the sleep condition, correlation between 2-back accuracy and FMD for the whole sample

*FMD, flow-mediated dilation; NIRS, near-infrared spectroscopy*.

In a double-blind crossover study, 30 healthy adults were administered chocolate drinks containing 520 and 994 mg flavanols and 46 mg matched control, with a 3-day washout between sessions (44). Cognitive assessment, designed to be cognitively fatiguing, consisted in a 10-min task battery administered in six consecutive repetitions, including a serial subtraction task and a rapid visual information processing task. Results showed that, compared to control, both 520 and 994 mg cocoa flavanols doses significantly improved working memory performance on serial subtractions.

Using a similar acute intervention, Field et al. (45) found that 2 ho after a 773-mg cocoa flavanols supplementation, young participants showed enhanced performance on the accuracy measure of a spatial working memory task, as well as performance improvements on a choice reaction time task. Also, visual function was improved, with better visual contrast sensitivity and time to detect motion direction (45).

By contrast, Pase et al. (46) failed to demonstrate any acute effect of 500 mg cocoa flavanols administration on cognition in older adults. Cognitive assessment included measures of memory and attention. However, as a limitation, participants had a standardized lunch break before testing session; it is therefore possible that flavonoids effects were masked by post-prandial factors (46).

Immediate cognitive enhancement induced by high-flavanol cocoa is generally supposed to be mediated by the capability of their metabolites to promote crucial changes in peripheral and central blood flow. Some investigations therefore included behavioral and cardiovascular measures to explore the association between acute post-intervention cognitive enhancement and changes in peripheral and central blood perfusion. In this respect, Massee et al. (47) showed improvements in a serial subtraction task after the acute administration, in healthy subjects, of a cocoa bar containing 250 mg of flavanols. However, this study failed to demonstrate significant concomitant cardiovascular improvements after treatment. Conversely, a pilot study in healthy young participants showed that a single higher dose of flavanol-rich cocoa (516 mg) caused a significant global increase in CBF 2 h post-treatment (31). This result suggests an increased brain perfusion mediated by NO-dependent vasodilation, after cocoa consumption (31, 49).

In line with this result, in a recent near-infrared spectroscopy study, the administration of 903 mg cocoa flavanols increased cerebral oxygenation during the Stroop task performance in comparison to placebo, again suggesting increased NO-mediated vasodilation. Conversely, cocoa flavanols had no acute effects on serum BDNF levels (48). Therefore, these data support the idea that flavanols-induced acute changes in brain perfusion could underpin immediate cognitive-enhancing effects. However, the increased cerebral blood oxygenation was not paralleled by concomitant effects on cognitive performance (48). The young age of participants may have contributed to the lack of behavioral effects. This could be particularly relevant in this study, as participant performed a very short, relatively simple cognitive task. Since cocoa has been shown to affect cognitive performance in healthy individuals undergoing sustained effortful cognitive processing (44), these results open the possibility that, at least in healthy young adults, a high level of cognitive demand is needed to uncover the subtle immediate behavioral effects of flavonoids.

Accordingly, it could be argued that flavanols may be more effective in sustaining performance in highly demanding contexts or in presence of impaired physiological functions (e.g., cardiovascular risk factors). In this respect, we recently evaluated, in healthy young subjects, the effects of acute flavanol-rich chocolate administration on cardiovascular and cognitive function known to be specifically impaired by lack of sleep (27, 28). To this purpose, participants underwent two baseline sessions, performed after undisturbed nocturnal sleep, and two experimental sessions, after one night of total sleep deprivation. Subjects were randomly assigned to consume, 90 min before each testing session, flavonoid-rich dark chocolate (520 mg total flavanols) or flavonoid-poor chocolate (88.5 mg total flavanols). Cognitive assessment included the Psychomotor Vigilance Task, as a measure of behavioral alertness, and a 2-back working memory task. A single flavanol-rich cocoa administration was effective at counteracting the vascular impairment observed after a night of total sleep deprivation. Moreover, in the female subsample, cocoa flavanols also counteracted cognitive impairment specifically induced by sleep loss, leading to improvements in the accuracy measure of the working memory task. This result supports the idea of a specific effectiveness of cocoa flavanols in sustaining higher order cognitive performance during sustained effortful processing in healthy individuals. Interestingly, in the whole sample higher flow-mediated dilation levels, indicative of improved endothelial function, were related to better performance accuracy. Thus, although direct CBF measurements were not included, the results indicate that a single flavanol-rich chocolate administration could exert beneficial effects on cognitive performance through the capability to induce acute changes in peripheral and central blood flow *via* NO-dependent vasodilation.

Altogether, investigations on acute cognitive-enhancing effects of cocoa flavanols support the effectiveness of cocoa at sustaining cognitive performance, although with mixed results. Quantity and bioavailability of the consumed cocoa flavanols, together with the length and cognitive load of the cognitive assessment, represent crucial factors that may significantly impact on the experimental outcomes.

## Conclusion

In recent years, in the context of an increased interest in the modulatory effects of food constituents on human health, cocoa flavanols have been suggested to display a variety of beneficial biological actions, including neuroprotection and cognitive modulation.

At present, the limited number of studies investigating cocoa flavanols intake and cognitive performance has produced mixed results. Indeed, physiological responses to flavonoid supplementation such as vasodilation, both at peripheral and central levels, have been consistently replicated; conversely, cognitive findings are not as unequivocal. When trying to account for such discrepancies, several methodological differences should be considered in dose, form, and timeframe of the cocoa flavanols administration, as well as in length and cognitive load of the experimental tasks. All these variables could have a remarkable impact on physiological and behavioral results and are likely to partly explain discrepancies among results.

Nevertheless, the evidence accumulated so far suggests that cocoa flavanols administration can be effective at sustaining cognitive performance, leading to improvements in measures of general cognition, attention, processing speed and memory. Beneficial cognitive effects of regular flavanols intake, particularly in patients at risk, are presumably mediated by direct neuroprotective actions as well as improvements in cerebrovascular and metabolic functions. Furthermore, acute administration of cocoa flavanols could result in immediate cognitive-enhancing effect, sustaining performance particularly in cognitively demanding conditions, including fatigue and sleep loss.

Altogether, research on the effects of cocoa and chocolate on human cognition, although at its preliminary stage, converges at pointing to cocoa as a new interesting nutraceutical tool to protect human cognition and counteract different types of cognitive decline, thus encouraging further investigations. Future research should be addressed to the identification of sensitive experimental measures capable of detecting flavanol-induced subtle changes in cognitive performance. Moreover, the characterization of appropriate dose, timing, and form of flavanols intervention required to reach beneficial effects in different populations, as well as the inclusion of fully matched placebo controls, are needed. Undefined remain also the effects of both acute and chronic administration of cocoa flavanols on chronic sleep deprivation and shift work, situations that may have a pronounced clinical impact. To elucidate immediate and long-term neuromodulatory properties of cocoa, future investigations should be ideally designed to include neuroimaging techniques in conjunction with cognitive and physiological measures.

## Author Contributions

VS wrote the manuscript. VS and MF conceived and organized the structure of the review. DT contributed to write the first draft of the manuscript. DT, GD, LG, and MF contributed to the critical revision of the paper and approved the final manuscript for the publication.

## Conflict of Interest Statement

The authors declare that the research was conducted in the absence of any commercial or financial relationships that could be construed as a potential conflict of interest.

## Abbreviations

BOLD, blood oxygenation level-dependent; BDNF, brain-derived neurotrophic factor; CBF, cerebral blood flow; ERK, extracellular signal-regulated kinase; LTP, long-term potentiation; MAPK, mitogen-activated protein kinase; NIRS, near-infrared spectroscopy; NO, nitric oxide; PI3-kinase/Akt, phosphoinositide 3-kinase; SSVEP, steady-state visually evoked potentials.
